# Health-state valuations for pertussis: methods for valuing short-term health states

**DOI:** 10.1186/1477-7525-3-17

**Published:** 2005-03-21

**Authors:** Grace M Lee, Joshua A Salomon, Charles W LeBaron, Tracy A Lieu

**Affiliations:** 1 Center for Child Health Care Studies, Department of Ambulatory Care and Prevention, Harvard Pilgrim Health Care and Harvard Medical School, 133 Brookline Ave, 6^th ^floor, Boston, MA 02215, USA; 2 Division of Infectious Diseases, Children's Hospital Boston, MA, USA; 3 Department of Population and International Health, Center for Population and Development Studies, Harvard School of Public Health, Boston, MA, USA; 4 National Immunization Program, Centers for Disease Control and Prevention, Atlanta, GA, USA; 5 Division of General Pediatrics, Children's Hospital Boston, MA, USA

**Keywords:** pertussis, time trade-off, willingness-to-pay, short-term health-state

## Abstract

**Background:**

The incidence of reported adolescent and adult pertussis continues to rise in the United States. Acellular pertussis vaccines for adolescents and adults have been developed and may be available soon for use in the U.S. Our objectives were: (1) to describe patient valuations of pertussis disease and vaccination; and (2) to compare valuations for short-term and long-term health states associated with pertussis.

**Methods:**

We conducted telephone surveys with 515 adult patients and parents of adolescent patients with pertussis in Massachusetts to determine valuations of pertussis-related health states for disease and vaccination using time trade-off (TTO) and contingent valuation (CV) techniques. Respondents were randomized to complete either a short-term or long-term TTO exercise. Discrimination between health states for each valuation technique was assessed using Tukey's method, and valuations for short-term vs. long-term health states were compared using the Wilcoxon rank-sum test.

**Results:**

Three hundred three (59%) and 309 (60%) respondents completed and understood the TTO and CV exercises, respectively. Overall, respondents gave lower valuations (lower TTO and higher CV values) to avoid a given state for adolescent/adult disease compared to vaccine adverse events. Infant complications due to pertussis were considered worse than adolescent/adult disease, regardless of the method of valuation. The short-term TTO resulted in lower mean valuations and larger mean differences between health states than the long-term TTO exercise.

**Conclusion:**

Pertussis was considered worse than adverse events due to vaccination. Short-term health-state valuation is better able to discriminate among health states, which is useful for cost-utility analysis.

## Background

The incidence of reported pertussis continues to rise in the United States despite high levels of childhood vaccination [1,2]. Waning immunity is thought to contribute to the particularly steep rise seen among adolescents and adults over the past two decades [3,4]. Acellular pertussis booster vaccines have been developed already and recommended for use in several other countries including Canada, France, Germany, and Australia [5-7]. A combined booster (TdaP) also may become available soon for use in the U.S.

Recently completed clinical trials suggest that the booster may prevent cough illness related to pertussis among adolescents and adults [8,9]. Though such illness does not result in mortality in this age group, it can be prolonged and associated with significant complications such as pneumonia or urinary incontinence [10,11]. However, implementation of a vaccination program for adolescents and/or adults would carry a significant cost. Policymakers will need to decide whether or not to recommend use of a vaccine where the health benefits to adolescents and adults are reductions in short-term morbidity, rather than mortality, and the health risks include adverse events from vaccination. Thus, further information regarding the relative valuations by patients of different potential consequences should be considered. Quantifying patient preferences is relevant to decisions about allocation of limited resources and is needed to assess the cost-effectiveness of vaccination in comparison to other well-accepted health interventions [12].

Methods commonly used for measuring health-state valuations include contingent valuation (CV) and time trade-off (TTO) [13]. Contingent valuation is an economic approach to valuing different outcomes using monetary value (e.g. willingness-to-pay, WTP) as a common metric; for example, the relative amounts that individuals would pay to avoid one health state or another may be interpreted as measures of their strength of preferences for time spent in these different states. An advantage to this approach is that respondents may find it relatively easy to value short-term health states in monetary terms since they are accustomed to assessing the dollar value of goods and services in everyday transactions. However, some outcomes are difficult to quantify using contingent valuation, e.g. how much a person is willing to pay to avoid death. Additionally, CV may be subject to anchoring effects and income effects [14].

Another common approach to measuring the benefits and harms of health interventions relies on health-state utilities. Utilities measure a person's preferences for specific outcomes on a scale of 0 to 1, on which 0 typically represents a state equivalent to death while 1 represents the best imaginable health. The time trade-off method is one of several approaches used to assess health utilities. Using the TTO method, respondents are asked how much longevity they would be willing to give up, if any, to avoid living with a particular health outcome. Traditionally, TTO questions have been framed as giving up time to avoid a long-term or chronic health state [15]. However, for many common health problems, including those caused by infections, the duration of the relevant health states is limited, not permanent. A more realistic approach would be to frame these conditions as short-term health states.

We conducted a survey using TTO and CV methods to determine the health-state valuations of adult patients and parents of adolescent patients diagnosed with pertussis. We compared two alternative approaches to framing TTO questions, based on either short-term or long-term health states. We hypothesized that framing questions as short-term rather than as long-term health outcomes would allow for better discrimination between states.

## Methods

### Study participants

Structured telephone interviews were conducted with adult patients (≥ 18 years) and parents of adolescent patients (11–17 years) diagnosed with confirmed pertussis in Massachusetts from December 1, 2001 to January 31, 2003 [11]. There were 800 cases of confirmed pertussis among adolescents and adults during this time period, and 517 (65%) respondents completed the telephone interviews, although two were excluded because the wrong health-state valuation survey was administered. Interviews included questions about medical and non-medical costs of illness and questions regarding health-state valuations for pertussis disease and vaccination. There were no significant differences in age, gender or race/ethnicity between respondents and all confirmed cases during the enrollment period. The study was reviewed and approved by the Institutional Review Boards of Children's Hospital Boston, Harvard Pilgrim Health Care, Massachusetts Department of Public Health, and Centers for Disease Control and Prevention.

### Survey protocol

Descriptions of the health states were derived with input from 3 pertussis experts (Table 1). Adults were asked questions about themselves while parents were asked to respond in reference to outcomes in their adolescents. We also asked both adults and parents of adolescents to value the prevention of infant health states (respiratory complications, neurologic complications) due to pertussis. All surveys included open-ended TTO and CV questions; in other words, respondents were asked once about the maximum amount of longevity they would give up, or the maximum amount of money they would be willing to pay, to avoid the health outcome in question. We chose the open-ended format [16-19] due to the large number of items evaluated and for ease of administration by telephone. Additionally, prior methodologic work on open-ended CV techniques has demonstrated similar results to the commonly used but more intensive alternative involving dichotomous-choice questions.[18]

**Table 1:** Health-state descriptions for outcomes associated with disease and vaccination.

**Health states**	**Description**
Local reaction	A sore upper arm that is slightly red, swollen, and tender after receiving a vaccination
Systemic Reaction	Low-grade fevers, headache, body ache, and decreased energy after receiving a vaccination
Mild cough	Coughing attacks that last for 1–2 minutes at a time and occur up to 8–10×/day. These coughing attacks wake you up at night several times a week, but you otherwise feel well between coughing attacks.
Severe cough	A cough that is so frequent and severe that it causes vomiting at least several times a week, difficulty eating or drinking, and difficulty sleeping every night.
Pneumonia	A severe cough with high fevers, chills, fatigue, and shortness of breath
Respiratory complications (apnea and cyanosis)	A 1-month-old baby that has coughing episodes so hard that he/she stops breathing and turns blue for 10–15 seconds. These episodes happen 8 to 10 times a day and the baby needs to be hospitalized, but is completely healthy afterwards.
Neurologic complications (seizures and encephalopathy)	A 1-month-old baby with seizures or convulsions. The seizures cause brief periods of being unconscious and the baby's arms and legs shake. They can last for up to 5 minutes at a time and happen several times a day. The baby needs to be hospitalized, but is completely healthy afterwards.

For TTO questions, respondents were asked the maximum amount of time they would be willing to trade from the end of their lives to avoid a particular health outcome now. Adults were asked how much time they would give up from the end of their lives to avoid living in a particular health state themselves, while parents were asked how much time they would give up from the end of their own lives to avert the health state in the adolescent [17,20]. This approach was adopted after pre-testing the survey instrument with parents, who were more willing to answer questions about trading time from their own lives than from their children's lives. For infant health states, both sets of respondents were asked to give up time from the ends of their own lives to avoid a long-term or short-term health state in an infant.

Although TTO questions traditionally have been framed using permanent health states for valuations of chronic disease [15], the health states associated with pertussis and vaccination are limited, lasting anywhere from days to weeks. Thus, asking respondents to imagine that they had to live for the rest of their lives with an infection or vaccine adverse event is not realistic. In order to address this concern and to test the hypothesis that framing the question as a short-term health state would significantly alter the TTO response, we created 2 versions of the survey – one with short-term and one with long-term (permanent) health states. Long-term health states were described as lasting for the lifetime of the infant, adolescent, or adult. Short-term health states were described as lasting for a duration of 8 weeks for the infant, adolescent or adult. We chose a constant duration for the short-term health states to ensure consistent responses regarding rank order and comparability of health states. In order to determine which version of the survey the parent or adult would receive, we used a random number generator to assign interviews to respondents once they consented to participate in the study.

CV questions elicited the amount of money that a respondent would be willing to pay to avoid living in a particular health state for 8 weeks. We chose to frame the CV questions as short-term health states for both versions of the survey based on prior work [21,22]. Respondents were instructed not to consider any money lost from missed work or any co-payments that would be required.

Telephone interviews were conducted in English or Spanish using standardized forms. Some respondents either were unable to complete or refused to answer the entire set of TTO questions or CV questions. If any answers were missing within a set of questions, respondents were excluded from analyses (of that set) in order to assess population means. If the respondent completed either set of questions, trained interviewers judged how well the respondent understood the TTO or CV questions separately based on a 3-point scale (good understanding, some understanding, limited understanding). Respondents were excluded from further analyses if they were thought to have either some or limited understanding of the tasks presented [23].

### Calculation of utilities – long-term states

**Figure 1:**
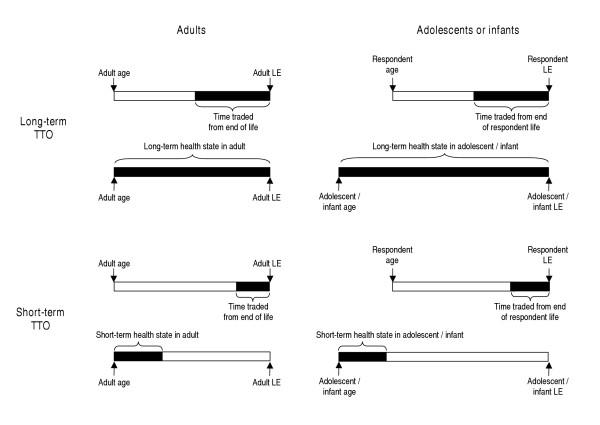
Conceptual model for calculating utilities for long-term and short-term health states for adults and adolescents or infants. In each of the four panels, the top bar indicates the amount of longevity that is given up from the end of the respondent's life in each of the trade-offs, and the bottom bar indicates the averted duration of time in a given health state. Abbreviations: TTO, time trade-off; LE, life expectancy.

We calculated utilities based on the TTO exercise, under alternative assumptions about discounting of future health outcomes. For long-term health states, the utility was based on the proportion of time that the respondent would be willing to give up to avoid a lifetime health state for themselves, for their adolescent child, or for a hypothetical infant (Figure 1). Life expectancy (LE) was calculated using age- and sex-specific cohort life tables [24]. In the cases of adolescents and infants, since the time given up would come from the adult respondent's lifespan, while the healthy time gained would accrue to the adolescent or infant, the computations required LE estimates for both the respondent and the beneficiary in the trade-off.

It is most straightforward computationally to start with the disutility, rather than the utility, of a particular health state, computed as the ratio of the duration of life that would be given up (to avoid the lifetime health state) to the expected duration of time lived in the health state. In the absence of discounting, the disutility was calculated simply by dividing the amount of time traded from the end of the respondent's life by the LE of the beneficiary. The utility was then calculated by subtracting this result from one. With discounting, we assumed that individuals compared the present values of the two different streams of life in the trade-off, in a way that reflects declining relative weight for future consequences, and we computed utilities based on a discount rate (r) of 3% per year [12]. As empirical studies on time preference have reported a range of discount rates [25-27], we also examined the sensitivity of our findings to alternative assumptions about discounting, including discount rates of 5% and 10% (as well as the 0% rate implied by the no-discounting case).

Using the formula for discounting a continuous stream of life [28], we obtained the present value of future time traded from the end of life (the numerator in the disutility calculation) by taking the difference between the discounted stream of normal life expectancy for the respondent and the discounted stream of shortened life expectancy:

(1/r) * (1 - e^-r (LE of respondent)^) - (1/r) * (1 - e^-r (LE of respondent - years of life traded)^)

The present value of the current life expectancy for the beneficiary (the denominator) was

(1/r) * (1 - e^-r (LE of beneficiary)^)

The disutility for a given health state was computed as the ratio of the two quantities, as in the undiscounted case, and the utility was computed by subtracting the ratio from one. For adult valuations, the respondent and beneficiary were the same. For parents of adolescents or for respondents considering a hypothetical infant, the numerator was based on years traded from the respondent's life, while the denominator was based on the life expectancy of the adolescent or infant.

### Calculation of utilities – short-term states

Utilities were calculated for the short-term health states in an analogous fashion, except that time from the end of the life of the respondent was traded to avoid 8 weeks of illness in the present time for the respondent, for the adolescent child, or for a hypothetical infant (Figure 1). The numerator was calculated in the same way as for the long-term states, assuming a 3% discount rate in the baseline analysis (and alternatives in sensitivity analysis):

(1/r) * (1 - e^-r (LE of respondent)^) - (1/r) * (1 - e^-r (LE of respondent - years of life traded)^)

For the denominator, discounting would have minimal impact because the duration considered is only 8 weeks and begins at the present, but we nevertheless converted this duration to its present value for consistency:

(1/r) * (1 - e^-r (8/52)^)

Again, the disutility was the ratio of these two quantities, and the utility was computed by subtracting the ratio from one.

### Statistical analysis

Utilities and WTP values are presented as means (with standard deviations) and medians (with interquartile ranges). We assumed that the maximum amount of discounted time traded from the end of the respondent's life could not exceed the duration of the present health state; thus, any utilities that would be negative based on the computations described above were instead set to 0. For parent respondents who were asked questions about how much time they would trade to avoid long-term health states in their adolescents, we used interval regression with left censoring to calculate mean utilities [29]. In interval regression, when parents were willing to trade off their full life expectancy to avoid a lifetime health outcome in their child, we treated this observation as providing only partial information about the amount that parents would give up, since they were limited by their life span – which was always shorter than the lifespan of the beneficiary adolescent. These observations were assumed to indicate a range of time spanning between the longevity of the parent and that of the adolescent. Interval regression was used to limit bias as a result of this constraint. When parents traded-off less than their full life expectancy, interval regression was equivalent to ordinary least squares regression. For infant health states, an analogous approach based on interval regression was applied.

To compare demographic characteristics of respondents for the short-term vs. long-term TTO surveys, we used the chi-squared test for categorical variables and the t-test for continuous variables. To determine if mean health state utilities and WTP values were significantly different from one another, we used Tukey's method, which is a non-parametric test that allows for multiple pairwise comparisons assuming all sample sizes are equal [30] Comparison of utilities for short-term vs. long-term health states was performed using the Wilcoxon rank sum test based on 2 independent samples [30]. Spearman rank correlation was used to determine associations between demographic characteristics and TTO or CV responses [30]. A p-value of <0.05 was considered statistically significant.

## Results

### Respondent characteristics

**Figure 2:**
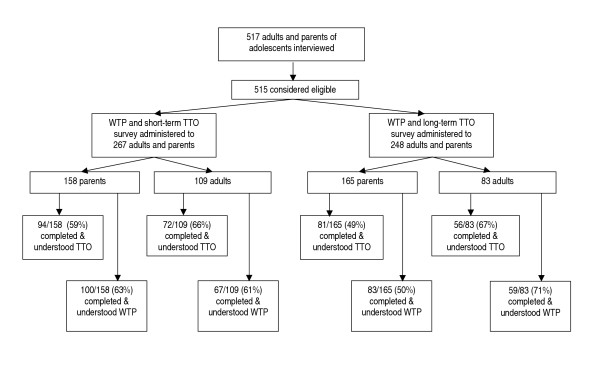
Study enrollment. Percentages indicate proportion of respondents who were given the survey that completed the entire set of questions and understood the TTO and WTP exercises. Abbreviations: TTO, time trade-off; WTP, willingness to pay.

Five hundred fifteen adult pertussis patients and parents of adolescent pertussis patients were eligible and participated in the survey (Figure 2). Characteristics of the respondents are described in Table 2. There were no significant differences between respondents who received either form of the survey. Overall, 303 (59%) respondents completed and understood the TTO portion of the survey and 309 (60%) respondents completed and understood the CV portion of the survey. When response rates of parents and adults were compared, we found no significant differences in response rates to the short-term TTO questions (p = 0.28); however, adults were significantly more likely to respond than parents of adolescents to the long-term TTO questions (p = 0.006). Other respondents were not included for analysis because: (1) the TTO (27%) or CV (22%) survey was not completed by respondents; or (2) one or more answers within a set of TTO (9%) or CV (12%) questions were not completed; or (3) respondents completed but were thought not to understand the TTO (6%) or CV (5%) exercise.

**Table 2:** Characteristics of respondents interviewed using short-term TTO (N = 267) vs. long-term TTO (N = 248).*

**Characteristics**	**Short-term TTO**	**Long-term TTO**	**P-value**
Mean age of respondent [range]**	42.4 [18–87]	41.7 [18–81]	0.49
Gender of respondent			
Female	207 (78%)	205 (83%)	0.35
Male	56 (21%)	40 (16%)	
Not available	4 (2%)	3 (1%)	
Race/ethnicity of respondent:			
White	238 (89%)	219 (88%)	0.67
Black	7 (3%)	4 (2%)	
Hispanic	11 (4%)	15 (6%)	
Other or unknown	11 (4%)	10 (4%)	
Educational level of respondent:			
Up to high school	67 (25%)	52 (21%)	0.48
Up to college or technical school	136 (51%)	143 (58%)	
>College	60 (22%)	49 (20%)	
Refused to answer	4 (2%)	4 (2%)	
Annual household income:			
<$20,000	28 (10%)	26 (10%)	0.79
$20,000–49,999	55 (21%)	53 (21%)	
$50,000–79,999	52 (19%)	58 (23%)	
≥ $80,000	98 (37%)	80 (32%)	
Refused to answer	34 (13%)	31 (13%)	

*Numbers may not add up to 100% due to rounding

**Missing ages for 8 parents of adolescents

We compared demographic characteristics of respondents who completed and understood the survey with those who did not. Parents of adolescents with higher household incomes (p = 0.022) and higher educational levels (p= 0.017) were more likely to complete and understand the CV survey. Also, parents who were white (p = 0.011) with higher educational levels (p = 0.010) were more likely to complete and understand the TTO survey. Adult respondents who completed and understood the CV and TTO survey were significantly younger (p = 0.006 for CV; p = 0.025 for TTO) and had higher educational levels (p = 0.005 for CV; p = 0.012 for TTO) than those who did not.

### Adolescent health states

**Figure 3:**
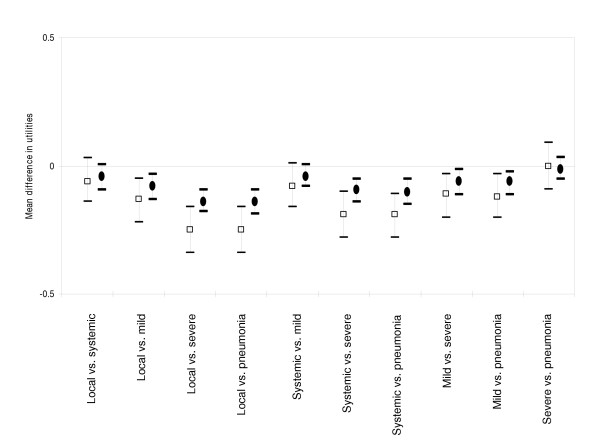
Mean difference between TTO utilities and 95% confidence intervals for short-term (squares) and long-term (circles) health states for adolescents.

CV and TTO responses for short-term and long-term health states for adolescents are described in Table 3. Based on mean utilities, parents of adolescents ranked the following long-term health states from best to worst: local reaction, systemic reaction, mild cough, severe cough, and pneumonia. Short-term health state rankings were similar, except mean utilities for severe cough and pneumonia were equivalent. For both short-term and long-term health states (Figure 3), we found significant differences in mean utilities (zero not included in the confidence interval) for most pairwise comparisons (Tukey's method, p<0.05). However, the mean utilities for health states that ranked close to each other were not significantly different, such as local reaction vs. systemic reaction, systemic reaction vs. mild cough, and severe cough vs. pneumonia. CV responses reflected rankings similar to TTO responses for parent respondents (Table 3). However, there were no significant differences between the amounts individuals were willing to pay to avoid adolescent health states.

**Table 3:** Adolescent Pertussis – days or years traded, utilities, and willingness-to-pay to avoid health states

	*Vaccination health states*		*Disease health states*		
	**Local reaction**	**Systemic reaction**	**Mild cough**	**Severe cough**	**Pneumonia**
**Short-term TTO (N = 94)**					
**Days traded**					
Mean (SD)	17 (46)	29 (61)	55 (117)	90 (162)	79 (114)
Median [25%–75%]	2 [0–14]	7 [2–28]	25 [7–56]	45 [14–56]	41 [14–70]
Utilities*					
Mean (SD)	0.92 (0.19)	0.86 (0.23)	0.78 (0.27)	0.67 (0.33)	0.67 (0.33)
Median [25%–75%]	0.99 [0.93–1.0]	0.96 [0.85–0.99]	0.87 [0.72–0.96]	0.78 [0.61–0.92]	0.78 [0.61–0.91]
**Long-term TTO (N = 81)**					
**Years traded**					
Mean (SD)	2.6 (4.1)	5.5 (5.9)	8.0 (6.7)	11.6 (9.0)	12.0 (9.5)
Median [25%–75%]	1 [0.1–5]	5 [1–5]	5 [5–10]	10 [5–20]	10 [5–20]
Utilities*					
Mean (SD)	0.97 (0.07)	0.93 (0.10)	0.89 (0.12)	0.83 (0.17)	0.82 (0.17)
Median [25%–75%]	0.99 [0.96–1.0]	0.95 [0.92–0.99]	0.93 [0.87–0.95]	0.88 [0.78–0.94]	0.88 [0.76–0.94]
**Willingness-to-pay (N = 183)**					
Mean (SD)	$18 (58)	$61 (174)	$3,003 (15,889)	$3,981 (16,797)	$4,265 (16,860)
Median [25%–75%]	$3 [1–13]	$13 [6–38]	$300 [150–1,500]	$750 [225–1,500]	$750 [263–1,500]

*Utilities were calculated assuming the maximum amount of time traded could not exceed the duration of the health state in the adolescent and assuming a discount rate of 3%.

### Adult health states

**Figure 4:**
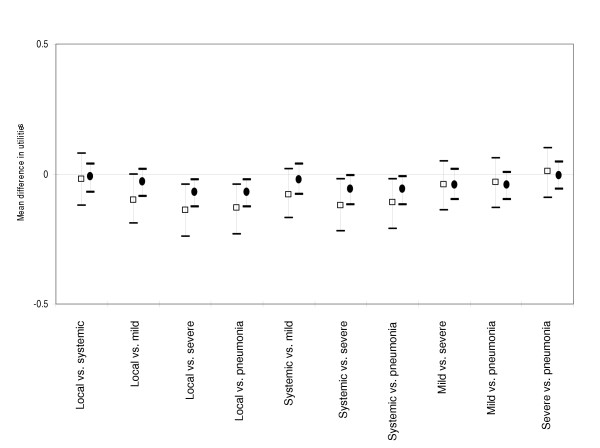
Mean difference between TTO utilities and 95% confidence intervals for short-term (squares) and long-term (circles) health states for adults.

Short-term and long-term TTO responses for adult respondents are described in Table 4. Based on mean utilities, adults ranked short-term health states in the following order: local reaction, systemic reaction, mild cough, pneumonia, and severe cough. Mean rankings for long-term health states were similar, except pneumonia and severe cough were equivalent. For short-term health states (Figure 4), mean differences in utilities were significantly different for 5 out of 10 pairwise comparisons (Tukey's method, p < 0.05). However, the only significant differences in utilities for long-term health states were: local reaction vs. severe cough, local reaction vs. pneumonia, systemic reaction vs. severe cough, and systemic reaction vs. pneumonia (Tukey's method, p < 0.05). CV responses again reflected a rank order similar to the TTO exercise (Table 4). We were unable to detect significant pairwise differences in the WTP amounts to avoid adult health states.

**Table 4:** Adult pertussis – days or years traded, utilities and willingness-to-pay to avoid health states

	*Vaccination health states*		*Disease health states*		
	**Local reaction**	**Systemic reaction**	**Mild cough**	**Severe cough**	**Pneumonia**
**Short-term TTO (N = 72)**					
**Days traded**					
Mean (SD)	24 (135)	26 (130)	80 (366)	99 (446)	101 (448)
Median [25%–75%]	0 [0–2]	2.5 [0–7]	8.5 [0.5–28]	14 [2–56]	14 [2–49]
Utilities*					
Mean (SD)	0.95 (0.18)	0.93 (0.18)	0.85 (0.26)	0.81 (0.30)	0.82 (0.30)
Median [25%–75%]	1.0 [0.99–1.0]	0.99 [0.95–1.0]	0.96 [0.88–1.0]	0.95 [0.81–0.99]	0.96 [0.83–0.99]
**Long-term TTO (N = 56)**					
**Years traded**					
Mean (SD)	0.4 (0.8)	1.4 (2.2)	2.7 (3.4)	4.7 (6.3)	4.7 (6.2)
Median [25%–75%]	0.03 [0–0.9]	0.8 [0.04–1.5]	1 [0.6–4.5]	2 [1–5]	2 [0.8–5]
Utilities*					
Mean (SD)	0.995 (0.01)	0.98 (0.03)	0.96 (0.06)	0.92 (0.14)	0.92 (0.16)
Median [25%–75%]	1.0 [0.99–1.0]	0.99 [0.98–1.0]	0.99 [0.96–1.0]	0.97 [0.92–0.99]	0.97 [0.91–0.99]
**Willingness-to-pay (N = 126)**					
Mean (SD)	$8 (17)	$41 (78)	$3,249 (14,062)	$4,141 (15,409)	$8,748 (66,907)
Median [25%–75%]	$3 [0–9]	$13 [6–38]	$450 [150–1,200]	$750 [300–1,500]	$750 [300–1,500]

*Utilities were calculated assuming the maximum amount of time traded could not exceed the duration of the health state in the adult and assuming a discount rate of 3%.

### Infant health states

TTO and CV responses for infant health states are described in Table 5. We asked all respondents to imagine they had a 1-month-old infant who developed pertussis that could result in either short-term (8 weeks followed by perfect health) or long-term health states. Mean utilities for short-term infant health states, such as respiratory or neurologic complications due to pertussis, were lower than mean utilities for vaccine adverse events or adolescent/adult disease. However, mean utilities for long-term infant health states were not significantly different from adolescent/adult disease utilities. All respondents were willing to pay significantly more to avoid infant disease compared to vaccine adverse events or adolescent/adult disease. Neurologic disease was considered significantly worse than infant respiratory disease regardless of the method of valuation.

**Table 5:** Infant pertussis – days or years traded, utilities, and willingness-to-pay to avoid health states*

	*Infant health states*	
	**Infant respiratory complications**	**Infant neurologic complications**
**Short-term TTO (N = 166)**		
**Days traded**		
Mean (SD)	174 (360)	226 (431)
Median [25%–75%]	56 [28–168]	56 [28–183]
**Utilities**		
Mean (SD)	0.58 (0.37)	0.51 (0.38)
Median [25%–75%]	0.72 [0.10–0.88]	0.64 [0.0–0.85]
**Long-term TTO (N = 137)**		
**Years traded**		
Mean (SD)	12.3 (10.9)	15.2 (12.3)
Median [25%–75%]	10 [5–20]	10 [5–20]
Utilities**		
Mean (SD)	0.82 (0.21)	0.77 (0.25)
Median [25%–75%]	0.89 [0.75–0.96]	0.87 [0.69–0.95]
**Willingness-to-pay (N = 309)**		
Mean (SD)	$13,016 (52,443)	$19,426 (61,074)
Median [25%–75%]	$1,500 [750–7,500]	$3,000 [750–10,000]

*Responses from adults and parents of adolescents were pooled.

**Utilities were calculated assuming the maximum amount of time traded could not exceed the duration of the health state assuming a discount rate of 3%.

### Comparison of utilities for short-term vs. long-term health states

Overall, mean utilities were higher for long-term health states than for short-term health states. These differences were significant for adolescents with mild cough (p = 0.045), severe cough (p = 0.001), and pneumonia (p = 0.001). No significant differences were found between short-term and long-term health states for adults. For infants, we also found significant differences with higher mean utilities reported for long-term health states compared to short-term health states (p < 0.001 for respiratory complications; p < 0.001 for neurologic complications).

### Association between demographic variables and TTO or WTP estimates

We evaluated associations between demographic characteristics such as age, race/ethnicity, education, and household income and estimates provided by respondents for the TTO or CV exercise. Older age was associated with lower utilities for short-term health states for adolescent/adult disease (i.e. mild cough, severe cough, pneumonia) and infant disease (i.e. respiratory and neurologic complications) (p < 0.05). Older age was also associated with lower utilities for long-term health states such as systemic reaction, adolescent/adult disease, and infant disease (p < 0.05). Higher income was associated with lower utilities for long-term mild cough and short-term respiratory complications, although older respondents were more likely to report higher household incomes (p < 0.001).

Higher income was significantly associated with higher WTP values for the following health states: systemic reaction, adolescent/adult disease, and infant disease (p < 0.05). We also found an association between higher respondent education and higher WTP estimates for pneumonia, although we note that education and income were themselves positively correlated (ρ = 0.358, p < 0.0001).

### Alternative assumptions about the discount rate

Tables 6A and 6B describe estimated mean utilities for short-term and long-term health states associated with disease and vaccination in adolescents, adults, and infants. At higher discount rates, the mean differences between utilities for different health states became smaller, regardless of age group or method of valuation.

**Table 6:** Utilities based on alternative discount rates of 0%, 5%, and 10%, for (A) adolescents, adults, and (B) infants. Utilities were calculated assuming the maximum amount of time traded could not exceed the duration of the health state

**A. Adolescents and adults**					
	*Vaccination health states*		*Disease health states*		
	**Local reaction**	**Systemic reaction**	**Mild cough**	**Severe cough**	**Pneumonia**
**Mean (SD) adolescent utilities for short-term TTO (N = 94)**					
0%	0.80 (0.32)	0.68 (0.36)	0.51 (0.39)	0.35 (0.38)	0.35 (0.37)
5%	0.95 (0.14)	0.92 (0.16)	0.87 (0.22)	0.80 (0.28)	0.80 (0.26)
10%	0.99 (0.03)	0.99 (0.04)	0.97 (0.08)	0.96 (0.11)	0.96 (0.08)
**Mean (SD) adolescent utilities for long-term TTO (N = 81)**					
0%	0.96 (0.06)	0.92 (0.09)	0.88 (0.10)	0.82 (0.13)	0.82 (0.14)
5%	0.97 (0.07)	0.94 (0.11)	0.91 (0.12)	0.85 (0.18)	0.85 (0.17)
10%	0.99 (0.05)	0.97 (0.09)	0.95 (0.11)	0.91 (0.17)	0.91 (0.15)
**Mean (SD) adult utilities for short-term TTO (N = 72)**					
0%	0.91 (0.24)	0.83 (0.29)	0.67 (0.38)	0.58 (0.42)	0.62 (0.40)
5%	0.97 (0.13)	0.96 (0.14)	0.90 (0.22)	0.88 (0.23)	0.88 (0.25)
10%	0.99 (0.04)	0.99 (0.04)	0.97 (0.07)	0.97 (0.08)	0.97 (0.08)
**Mean (SD) adult utilities for long-term TTO (N = 56)**					
0%	0.99 (0.02)	0.97 (0.06)	0.93 (0.09)	0.88 (0.17)	0.88 (0.18)
5%	1.0 (0.01)	0.99 (0.03)	0.97 (0.05)	0.94 (0.13)	0.94 (0.15)
10%	1.0 (0.00)	1.0 (0.01)	0.99 (0.02)	0.97 (0.09)	0.96 (0.12)
**B. Infants**					
	*Infant health states*				
	**Infant respiratory complications**	**Infant neurologic complications**			
			
**Short-term TTO (N = 166)**					
0%	0.27 (0.36)	0.21 (0.33)			
5%	0.71 (0.35)	0.66 (0.36)			
10%	0.92 (0.17)	0.90 (0.19)			
**Long-term TTO (N = 147)**					
0%	0.36 (0.18)	0.33 (0.19)			
5%	0.84 (0.21)	0.78 (0.26)			
10%	0.89 (0.20)	0.84 (0.27)			

## Discussion

Vaccination programs in the US have traditionally been life-saving and cost-saving [31,32]. However, the focus of newer vaccines being developed has shifted from preventing mortality to preventing morbidity. In this situation, the risks of vaccine adverse events need to be weighed carefully against their benefits. Health-state valuation studies are useful to assess the relative risks and benefits of potential future vaccination programs under consideration in the US. Our study examined valuations associated with adolescent/adult pertussis disease and vaccination. Overall, respondents rated adolescent and adult pertussis as worse than vaccine adverse events. Also, infant complications due to pertussis were ranked as worse than adolescent/adult disease.

We explored differences in utilities for short-term and long-term health states using open-ended TTO questions. Other methods for short-term health state valuation described in the literature include chained TTO, sleep tradeoff (STO), and waiting tradeoff (WTO). The chained TTO has been shown to have good consistency and reliability [13,33,34]. However, the chained procedure involves an extra step and may result in a significant cognitive burden due to the complexity of the task. The sleep tradeoff asks people how much time they would be willing to sleep in a non-refreshing/non-dream state to avoid living with a short-term health problem [35,36]. Unfortunately, this method may not be appropriate for valuing health states associated with pertussis since sleep disturbance occurs in a majority of infected individuals, which may confound responses regarding sleep [11]. The waiting tradeoff proposed by Swan *et al. *is an alternative approach for assessing process utility [37]. While this approach is clearly useful for situations that involve diagnostic procedures, its applicability to other short-term health states such as infections is limited. We felt the open-ended format was the most appropriate method for our study population given the limitations of alternatives and due to improved ease of administration by telephone.

We found that the rankings of health states based on mean utilities were essentially the same using either short-term or long-term health states. However, the short-term TTO resulted in lower mean utility estimates and larger mean differences between health states than the long-term TTO exercise, thus allowing for better discrimination of health states in cost-utility analyses. These results suggest that responses may not fulfill the constant proportional trade-off assumption, which requires that the TTO utility be independent of the duration of the specified health state. While previous studies using TTO and other valuation methods have also found that the constant proportional trade-off assumption does not always hold, the direction of the discrepancy has been mixed [38-40].

The short-term health state approach may have violated the constant proportional trade-off assumption because respondents were less averse to giving up small amounts of time from the ends of their lives (days or weeks) compared to large amounts of time (months or years) in the long-term approach. In other words, asking respondents to give up a few days or weeks from the ends of their lives may not be considered a significant loss, even to avoid a short-duration health state lasting only 8 weeks. However, giving up months or years of life is considerably more difficult for individuals, even to avoid an intermediate- or long-term health state. It may be that a threshold exists whereby individuals are more willing to give up a very small portion of their lives for perfect health, but as the duration of health states increase, they are less willing to give up time per health unit gained, resulting in failure to behave according to the constant proportional trade-off assumption. This aversion to giving up larger amounts of time may play an important role in measuring utilities, particularly since the short duration of these health states over the lifetime of individuals would otherwise lead to nearly imperceptible, but arguably important differences in terms of quality-adjusted life years.

The impact of discounting is important to consider since assuming no discounting can lead to a downward bias, while a high discount rate can lead to an upward bias, which may strongly devalue the benefits of any preventive intervention [12,41-43]. We assumed a 3% discount rate in our baseline analysis, though there is no clear standard regarding the optimal discount rate for societal decisions. We also examined the implications of varying the discount rate between 0 and 10%. If we assumed no discounting of health preferences over time, the mean utilities for all health states were lower and spread over a wider range. At a discount rate of 10%, the mean values for all health states approached 1.0 and mean differences between health states were much smaller. Further empirical investigation of societal discount rates for prevention programs is needed.

The CV exercise resulted in similar mean health state rankings to our TTO exercise, and these estimates were positively correlated with income, which is not surprising since individuals were asked to respond in consideration of their actual household income [44]. For the TTO, we found an inverse association between age and utility estimates. This finding is consistent with previous studies that have shown older individuals provide lower utility estimates for health states [45,46]. It may be that increased awareness of the reality of living in poor health is better understood by older respondents [46].

As always, there are limitations to our study. First, our respondents were either adult pertussis patients or parents of adolescents with pertussis. We elicited patient and caregiver valuations as part of a larger study to determine societal costs of pertussis in adolescents and adults. While the U.S. Panel on Cost-effectiveness in Health and Medicine suggests that community preferences be used where possible[12], there is ongoing debate over whose preferences should be included in cost-effectiveness analyses.[47] There are certain practical advantages to surveying patients and caregivers. Because these individuals had recent first-hand experience with the disease, reasonably short descriptions could be used to sufficiently characterize a series of health states associated with pertussis, making administration by telephone feasible. While there is no perfect measure of health, patient preferences can help to inform societal values and should be given further consideration. If community valuations are collected in subsequent studies, it will be useful to compare them to the patient and caregiver valuations collected here.

Second, selection bias might arise from our survey completion rate of around 60%, although this is comparable to other published valuation studies [20,23]. In our study, a significant proportion of respondents did not complete the survey (22–27%), refused to answer the entire set of questions (9–12%), or did not understand the exercise (5–6%). We believe this may have been due in part to respondent burden, because preference questions were asked at the end of a lengthy cost interview. Also, we asked both WTP and TTO questions for 7 separate health states. In a separate analysis that included all answers to the set of questions regardless of the level of understanding, we found that the rank order of health states remained consistent, which is reassuring.

Because of the complexity of the task required, it is not surprising that respondents with higher educational levels were more likely to complete and understand the preferences exercise. In addition, most respondents were white, well educated, and had relatively high household incomes. Household income was associated with utilities as well as willingness-to-pay to avoid pertussis. To address this limitation, economic analyses of pertussis should vary willingness-to-pay and utilities over wide ranges that would reflect the preferences of a general population. Further research in more socioeconomically diverse populations should also be considered.

Another issue that should be explored more thoroughly is the impact of parents as surrogate respondents for children and the method of preference elicitation. We asked parents of adolescents to serve as proxy respondents for their child. Interestingly, parents of adolescents were less likely to provide answers to the long-term TTO exercise than adult respondents, which may suggest that parents had difficulty answering the TTO question for long-term illnesses in their children. Also, trading time from the parent's life to avoid illness in a child may result in preferences that incorporate other aspects of their relationship such as altruism. Previous work in the CV literature has suggested that altruism may significantly affect valuations. For example, Liu *et al. *found that a mother's WTP to prevent a cold is approximately twice as large for the child as for the mother[21]. While parents are often considered to be the health care decision makers for their child, further work on eliciting health state valuations directly from children would provide useful information. In addition, we asked parents how much time they would be willing to give up from the end of their lives to avoid illness in their child because we found that some parents refused to trade time from their child's life whereas they were willing to trade from their own life. Though this did not affect our calculation of short-term utilities (since a common denominator of 8 weeks was used), it did affect our calculation of long-term utilities where the denominator was based on the life expectancy of the child.

## Conclusion

In this study, we estimated health-state valuations regarding pertussis disease and vaccination among adult patients and parents of adolescent patients. Patient preferences in conjunction with health outcomes will be key factors in deciding whether or not to implement a universal vaccination policy for adolescents or adults. The results from our study suggest that short-term health-state valuation may provide a reasonable approach to assessing preferences given its superior ability to discriminate between states, which may be particularly useful for cost-utility analyses for future vaccination programs.

## Authors' contributions

Dr. Lee had full access to all of the data in the study and takes responsibility for the integrity of the data and the accuracy of the data analysis.

*Study concept and design: *Lee, Salomon, Lieu

*Acquisition of data: *Lee, Lieu

*Analysis and interpretation of data: *Lee, Salomon, LeBaron, Lieu

*Drafting of the manuscript: *Lee

*Critical revision of the manuscript for important intellectual content: *Lee, Salomon, LeBaron, Lieu

*Statistical analysis: *Lee, Salomon

*Obtained funding: *Lieu

*Administrative, technical, or material support: *Lieu

*Study supervision: *Salomon, Lieu

## Financial support

This study, part of the Joint Initiative on Vaccine Economics Project, was supported by the National Immunization Program, Centers for Disease Control and Prevention, via cooperative agreement with the Association of Teachers of Preventive Medicine, Task order #TS-0675. Dr. Lee's work was also supported in part by the grants T32 HS00063 and K08 HS013908-01A1 from the Agency for Healthcare Research and Quality, U.S. Department of Health and Human Services. Dr. Salomon was supported by the National Institute on Aging (Grant P01 AG17625).
